# Social relations and life satisfaction: the role of friends

**DOI:** 10.1186/s41118-018-0032-z

**Published:** 2018-05-04

**Authors:** Viviana Amati, Silvia Meggiolaro, Giulia Rivellini, Susanna Zaccarin

**Affiliations:** 1 Department of Humanities, Social and Political Sciences, ETH, Weinbergstr.109, 8092 Zürich, Switzerland; 2 Department of Statistical Sciences, Via C. Battisti, 241, 35121 Padua, Italy; 3 Department of Statistical Sciences, Catholic University, Largo Gemelli, 1, 20123 Milan, Italy; 4 Department of Economics, Business, Mathematics and Statistics, University of Trieste, P.le Europa 1, 34127 Trieste, Italy

**Keywords:** Social capital, Multipurpose survey, Friendship relationships, Life satisfaction

## Abstract

Social capital is defined as the individual’s pool of social resources found in his/her personal network. A recent study on Italians living as couples has shown that friendship relationships, beyond those within an individual’s family, are an important source of support. Here, we used data from *Aspects of Daily Life*, the Italian National Statistical Institute’s 2012 multipurpose survey, to analyze the relation between friendship ties and life satisfaction. Our results show that friendship, in terms of intensity (measured by the frequency with which individuals see their friends) and quality (measured by the satisfaction with friendship relationships), is positively associated to life satisfaction.

## Introduction

The concept of social capital and its analysis has attracted the attention of several disciplines (economics, sociology, psychology, etc.) in the past 40 years. Starting from the seminal works of Coleman (1988), a multitude of social capital definitions and conceptualizations has been proposed (e.g., Durlauf and Fafchamps 2005).

The main concept present in all of the current definitions is that social capital is a resource that resides in the networks and groups which people belong to, rather than an individual characteristic or a personality trait. Portes (1998) defined social capital as “the ability of actors to secure benefits by virtue of their membership in social networks or other social structures,” stressing that whereas “economic capital is in people’s bank accounts and human capital is inside their heads, social capital inheres in the structure of their relationships” (p. 7). Lin et al. (2001, p. 24) defined social capital as “resources embedded in a network, accessed, and used by actors for actions.”

The term “network” is used to describe the ties and social relationships in which an individual is embedded. A network is composed of a finite set of actors and the relations among them. There are two primary types of networks: complete and ego-centered. While complete networks describe the links between all members of a group, ego-centered networks are defined by “looking at relations from the orientation of a particular person” (Breiger 2004, p. 509), that is called ego, and therefore, ego-centered networks focus on an ego and his/her relations with a set of alters.

Recognizing the importance of identifying individuals’ networks to understand many phenomena (e.g., social support, socioeconomic mobility, social integration, health conditions), several national and international surveys (e.g., the Generations and Gender Surveys, the International Social Survey Programme and the European Quality of Life Survey, and the Italian Multipurpose surveys) provide information on the ego-centered network of each respondent. This data might be used to investigate network-based sources of social capital at individual level, even though these surveys are neither network-oriented nor social capital-oriented. Because of the availability of these broad surveys that measure both social relations and aspects of an individual’s life, more studies have considered the potential role of social networks in the life of individuals.

One branch of research has focused on the link between the characteristics and composition of social networks and the variety of support (emotional, material, and instrumental) available and/or received by individuals (Zhu et al. 2013; Amati et al. 2017). Another issue commonly considered in the literature is the influence of an individual’s social interactions on his or her behaviors, such as fertility choices (Bernardi et al. 2007; Keim et al. 2009). Finally, the role of social networks on an individual’s well-being has also been examined (Taylor et al. 2001; Haller and Hadler 2006; Powdthavee 2008).

The practical use of multipurpose surveys for the analysis mentioned above is clearly worthwhile. These types of surveys offer a large amount of information, allowing researchers to study the role of social capital in a variety of outcome variables controlling for individual and group-level characteristics. In the long term, repeated surveys might also provide longitudinal data for further investigation on whether social capital and its role in an individual’s life change over time. The data collected from general surveys can also be analyzed to provide hints on certain phenomena (e.g., quality of life, social and family life, lifestyle, friendship) when specific surveys are not available.

The current study supplements research that considers the role of resources embedded in a social network for an individual’s subjective well-being. In this paper, a particular facet of social capital is analyzed: the role of friends as alters in ego-centered networks (Breiger 2004). This choice stemmed from a recent study on Italians living in couple, which showed that friendship relationships are valuable sources of support (e.g., instrumental, emotional, and companionship) that supplement the support inherent in traditional or expected ties to parents and relatives (Amati et al. 2015). This paper examines the role of friends in an individual’s subjective well-being, which is measured by life satisfaction.

Data was obtained from the multipurpose survey “Aspects of Daily Life,” collected by the Italian National Statistical Institute (Istat) in 2012. The focus of the current study is on individuals aged 18–64 years old. This data allows investigation of friendship’s effects on life satisfaction, measuring in terms of the frequency with which individuals see their friends (intensity) and the satisfaction with friendship relationships (quality). The underlying hypothesis is that friendship relationships influence life satisfaction through the potential (instrumental and emotional) resources that friends may provide. Those resources depend on both the presence of friends (measured in terms of frequency of meeting friends) and on the quality of the friendship (friendship satisfaction).

The paper is organized as follows: the “Background” section provides a review of the studies that considered the link between friendship and life satisfaction, with particular attention on the importance of distinguishing friendship network characteristics in terms of intensity and quality of the relations with friends (“Quality and quantity in friendship relationships” section). Survey data and the strategy of analysis are described in the “Data and methods” section. Results are reported in the “Results” section and discussed in the “Concluding remarks” section.

## Background

### Social relations, friendship, and life satisfaction

Subjective well-being refers to the many types of evaluations that people make of their lives (Diener 2006) and is conceptualized and measured in different ways and with different proxies (Kahneman and Deaton 2010; Dolan and Metcalfe 2012).

Although life satisfaction is only one factor in the general construct of subjective well-being, it is routinely used as a measure of subjective well-being in many studies (e.g., Fagerstrӧm et al. 2007; Ball and Chernova 2008; Shields et al. 2009). In particular, life satisfaction, referring to a holistic evaluation of the person’s own life (Pavot and Diener 1993; Peterson et al. 2005), concerns the cognitive component of the subjective well-being. Another commonly used measure for subjective well-being is happiness (Diener 2006), often used interchangeably with life satisfaction.

There is substantial evidence in the psychological and sociological literature that individuals with richer networks of active social relationships tend to be more satisfied and happier with their lives. This positive role of social relationships on subjective well-being may be explained by the benefits they bring. First, relationships, being key players in affirming an individual’s sense of self, satisfy the basic human need for belongingness (Deci and Ryan 2002) and are a source of positive affirmation. The levels of subjective well-being increase with the number of people an individual can trust and confide in and with whom he or she can discuss problems or important matters. On the other hand, these levels decrease with a surplus presence of acquaintances or strangers in the network (see Burt 1987; Taylor et al. 2001; Powdthavee 2008).

Second, the presence of social relationships has positive impacts on mental and physical health, contributing to an individual’s general well-being, whereas the absence of social relationships increases an individual’s susceptibility to psychological distress (Campbell 1981; Nguyen et al. 2015). Several studies have shown that social relations stimulate individuals to fight diseases (Myers 2000) and reinforce healthy behaviors (Putnam 2000). Social interactions have the potential to protect individuals at risk (e.g., encouraging them to develop adjustment techniques to face the difficulties) and promote positive personal and social development, which diminishes the exposure to various types of stress (Myers 2000; Halpern 2005) and increases the ability to cope with it.

Finally, social relationships form a resource pool for an individual. These resources can take several forms, such as access to useful information, company (e.g., personal and intimate relationships, time spent talking together, and shared amusement time or meals), and emotional (e.g., advice about a serious personal or family matter) and instrumental (e.g., economic aid, administrative procedures, house-keeping) support. Several studies have detailed how receiving support contributes to higher well-being, although the effects may vary by the type and the provider(s) of support (Merz and Huxhold 2010). In a wider perspective, social relationships serve as buffers that diminish the negative consequences of stressful life events, such as bereavement, rape, job loss, and illness (Myers 2000). The perceived availability of support or received support from others may, indeed, lead to a more benign appraisal of a negative situation.

**Fig. 1:**
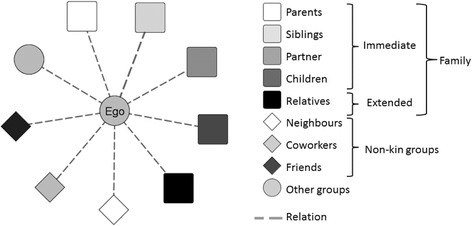
Ego and kinds of alters in an ego-centered network

In this view, friendships, considered as voluntary relationships that involve a variety of activities, may contribute significantly to the overall subjective well-being (Clark and Graham 2005). Friends are only one of the possible alters in an ego-centered network, as represented by Fig. 1. At the same time, they are the only alters that a person chooses as a node that belongs to his/her personal network while parents, siblings, and relatives are “the family you are born with”, and neighbors and coworkers are people an individual usually encounters in a preexisting situation, “friends are the family you choose” (Wrzus et al. 2012, p. 465).

As for many relationships, friendship strongly depends on meeting opportunities (Verbrugge 1977; Feld 1981), as determined by social settings (Pattison and Robins 2002), and the decision of individuals to establish a certain friendship tie. This indicates that friendship is often related to positive interpersonal relationships which are important and meaningful to an individual and satisfy various provisions (intimacy, support, loyalty, self-validation). In addition, support from friends is usually voluntary, sustained only by feelings of affection, mutuality, and love (Yeung and Fung 2007), but not motivated by moral obligations (typical of family ties, Merz et al. 2009).

Recent years witnessed the growth of social contexts where the importance of friends is increasing. First, sociodemographic changes, such as the reduction in the number of children in each family and a weakening of traditional communities like churches and extended families, raise the relevance of friends in the network (Suanet and Antonucci 2017). Second, family and marital relationships have also changed over the last few decades; through divorce and remarriage, they appear more complex and less robust. The breakup of the immediate household and of the extended family can have direct implications on the relationships among the household members. Friends can substitute, in a certain sense, the traditional family (Ghisleni 2012), offering invaluable advice, support, and companionship.

Only the positive consequences of friendship on well-being have been considered so far. However, friendships might also play a negative role for an individual’s well-being. Concerning the need for belongingness, some friends may be disturbed individuals and thus have a damaging effect on an individual (Halpern 2005); in addition, the fear of being criticized or excluded may also have a negative impact on well-being. As to the health motivation friends might encourage individuals toward unhealthy behaviors, such as smoking or overeating (Schaefer et al. 2012; Huang et al. 2014). Finally, unfulfilled expectations may negatively affect the benefits derived from support. Despite these potentially negative influences, friendships are generally expected to have a positive role in an individual’s well-being (Van Der Horst and Coffè 2012).

### Quality and quantity in friendship relationships

Friendship relationships can recall both quantitative and qualitative dimensions. For instance, asking about having or not having friendship ties is often related to the count of the number of friends; similarly, evaluating the degree of mutual concern and interest calls for a quantitative measure, such as the duration of friendship or the frequency of interaction. Distinguishing between best friends and friends, real or close friends, “really true” or “not true” friends (Boman IV et al. 2012) is qualitative measures of friendship relationships. The qualitative aspects are determined by the fact that friendship relations might be close, intense, and supportive at different levels. In general, the closer the friendship, the more evident the various qualitative attributes of friendship (Demir and Özdemir 2010).

The different definitions of friendship emphasize both the qualitative dimension and the interactive sphere of friendship. Alberoni (1984) defined friendship as “a clear, trusted, and confident feeling” (p.11). Hays (1988), based on a review of theoretical and empirical literature, suggested a more comprehensive definition of friendship, wherein “a voluntary interdependence between two persons over time, that is intended to facilitate socio-emotional goals of the participants, and may involve varying types and degrees of companionship, intimacy, affection, and mutual assistance” (p. 395). The Encyclopedia Britannica defines friendship as a “state of enduring affection, esteem, intimacy, and trust between two people” (Berger et al. 2017). All these definitions indicate that friendship is recognized as a dyadic relationship by both members of the relationship and is characterized by a bond or tie of reciprocated affection. It is not obligatory, carrying with it no formal duties or legal obligations to one another, and is typically egalitarian in nature and almost always characterized by companionship and shared activities (Berger et al. 2017).

The network perspective emphasizes the dyadic nature of friendship and stresses the quantitative dimension of friendship relationships in terms of the “strength” of an interpersonal tie, where “the strength of a tie is a (probably linear) combination of the amount of time, the emotional intensity, the intimacy (mutual confiding), and the reciprocal services which characterize the tie” (Granovetter 1973 p. 1361).

The analysis of the interaction between friendships and personal well-being or life satisfaction is strongly influenced by the available data, which often regards the quantitative dimension of friendships. Several studies have emphasized how this dimension affects an individual’s well-being through the benefits friendship brings. In particular, a large number of friends, as well as more contact with these friends and a low heterogeneity of the friendship network, are related to more social trust, less stress, and better health (McCamish-Svensson et al. 1999; Van Der Horst and Coffè 2012). From the point of view of support, having many friends and frequent contact with them increases the chance of receiving help when needed (Van Der Horst and Coffè 2012). More broadly, the frequency of meeting a friend can be an indicator of the strength or intensity of the relationship (Haines et al. 1996). Stronger relationships might imply increased knowledge of an individual’s needs, thus creating a stronger source of potential help. Regarding the qualitative dimension, empirical research is quite scanty; however, what is available shows that satisfaction with a friendship is strictly related to an individual’s well-being and life satisfaction (Diener and Diener 2009; Froneman 2014).

Taking into account both the questionnaire constraints and the research focus on studying the role of friends in life satisfaction, this study focused on adulthood and measured the quantitative dimension of friendship through the intensity of interaction (“frequency of meeting friends”) and the qualitative dimension through the satisfaction with friendship relationships. The hypotheses that the intensity of relations with friends might have a different effect depending on the level of satisfaction with these relations were tested. A faithful frequency of contacts with friends, together with positive satisfaction with friendship relationships, connects individuals to a range of extra benefits, including a higher sense of belongingness, better health, and more support (Van Der Horst and Coffè 2012).

## Data and methods

### The multipurpose survey “Aspects of Daily Life”

Data was drawn from the cross-sectional, multipurpose survey “Aspects of Daily Life,” carried out in Italy by Istat. Conducted annually since 1993, it is a large sample survey that interviews a sample of approximately 50,000 people in about 20,000 households. It collects information on several dimensions of life for each individual, including basic socio-demographic characteristics of individuals (age, sex, education) and of their households (socio-economic status and family structure) and information on health, lifestyle, religious practices, and social integration.

Starting in 2010, the survey investigated life satisfaction for individuals aged over 14, asking the following question: “How satisfied are you with your life on the whole at present?” Answers range between 0 (not satisfied at all) and 10 (very satisfied). These levels of life satisfaction represent a crude measurement of the underlying continuous variable, i.e., life satisfaction, which cannot be measured on a continuous scale.

**Fig. 2:**
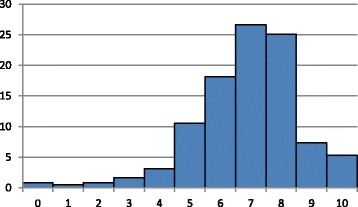
Percentage distributions of individuals aged 18–64 according to their life satisfaction

The current study focuses on the most recent survey data (2012) and considers the life satisfaction of 25,190 individuals (ages 18–64). Figure 2 reports the percentage distributions of these individuals according to their life satisfaction. It demonstrates that the proportions of individuals who declared indexes of life satisfaction under 5 are quite low; those with life satisfaction equal to 5, however, are not negligible. On the whole, only 17.5% of individuals declared a life satisfaction under 6. Most individuals (64.4%) seem to be quite satisfied in their life, declaring values equal to or greater than 7.

Next, to the question on life satisfaction, there are two additional questions collecting information on two different aspects of friendship relationships: the frequency at which individuals usually meet their friends in their leisure time and the satisfaction of individuals with friendship relationships over the previous 12 months. The first aspect can be seen as a proxy for the intensity of friendships interaction. Response options of the corresponding question consisted of 1 = every day, 2 = more than once per week, 3 = once per week, 4 = several times (but less than 4) per month, 5 = sometimes per year, 6 = never, and 7 = without friends. In the following analyses, these seven categories are grouped^1^ to distinguish individuals meeting their friends as follows: more than once a week (1, 2), once a week or several times a month (3, 4), and less often or not having friends (5, 6, 7).

The second question concerns satisfaction of individuals with friendship relationships understood as the quality of friendships. This satisfaction can be considered as a proxy for the quality of friendship. The corresponding question response options consisted of 1 = very satisfied, 2 = quite satisfied, 3 = not very satisfied, and 4 = not satisfied at all. In the following analyses, the last two categories are grouped together because of the low proportions of individuals indicating no satisfaction at all in friendships. Table 1 reports the distribution of individuals according to both of these key variables describing friendship. The data shows that most individuals meet friends more than once a week and are quite satisfied with the friendship relationship.

**Table 1:** Percentage distributions of individuals aged 18–64 according to their friendship relationships

	%
Frequency of meeting friends	
More than once per week	46.9
Once per week or several times a month	42.6
Sometimes per year or less often or without friends	10.5
Satisfaction with friendship relationships	
Very satisfied	27.4
Quite satisfied	60.7
Not satisfied	11.9
Total	25,190

Table 2 shows that friendship and life satisfaction are related. Individuals meeting their friends more often and declaring themselves more satisfied with their friendship relationships tend to have a higher life satisfaction when compared to people who rarely meet their friend and/or are not satisfied with their relationships. In addition, the association between these variables describing friendship relationships and life satisfaction is statistically significant (*χ*^2^ = 2288.2, df = 20, *p* value < 0.001 and *χ*^2^ = 394.04, df = 20, *p* value < 0.001, respectively, for friendship satisfaction and frequency of contacts). However, these associations may be due to other compositional factors. Younger individuals meet their friends more often than older ones, and literature has shown that life satisfaction is higher among younger people (Demir et al. 2015, Walen and Lachman 2000). Thus, the role of friendship has to be examined using multivariate analyses, while controlling for a series of other variables.

**Table 2:** Some descriptive indicators of an individual’s life satisfaction according to their friendship relationships

	Mean	% with satisfaction greater than or equal to 8	% with satisfaction greater than or equal to 7	% with satisfaction greater than or equal to 6
Frequency of meeting friends				
More than once per week	6.95	38.8	66.26	84.20
Once per week or several times a month	6.91	38.1	65.00	83.43
Sometimes per year or less often or without friends	6.39	31.8	53.46	71.23
Satisfaction with friendship relationships				
Very satisfied	7.44	53.5	78.01	88.91
Quite satisfied	6.80	34.0	62.34	82.56
Not satisfied	5.97	20.7	43.34	65.40

### Methods and strategy of analysis

A multilevel logistic regression model was estimated to investigate the relation between life satisfaction (dependent variable) and the frequency of meeting friends and the satisfaction of friendship relationships (explanatory variables), controlling for several covariates. The choice of a random intercept logistic regression model was motivated by both the data structure and the level of measurements of the dependent variable.

Specifically, the data shows a nested structure, where the first-level units are the individuals and the second-level units are the families. To control for the nested structure, we considered a multilevel model, rather than simply correcting the estimated standard errors for the presence of clustered units in the sample. The limited number of individuals belonging to the same family (the 99% of the families has a size smaller than four) might be problematic for the methods because of the correction of the standard errors (Leoni 2009).

Regarding the dependent variable, the fact that it is measured on an ordinal scale should be considered. Several models have been proposed for the analysis of ordinal variables, among them the ordinal logistic regression model (Agresti 2010). This model is the extension of the multinomial logit model to ordinal variables. One of the fundamental assumptions underlying the ordinal logistic regression model is the proportional odds assumption, requiring that the relationship between each pair of outcome categories is the same. When this assumption is violated, the estimates might be biased and the standard errors might be either underestimated or overestimated, leading to misleading conclusions derived from ordinal regression models. An alternative is available in the partial ordinal logistic regression model which relaxes the assumption of proportional odds, allowing the parameters to vary across the level of the dependent variables, but yielding a less parsimonious model.

The analyzed data provides evidence against the assumption of proportional odds (*χ*^2^ = 4456, df = 414, *p* value < 0.001); therefore, a partial ordinal logistic regression would be adequate. However, the number of categories of the dependent variable is far from negligible, and estimating such a model would yield a non-parsimonious model that is difficult to be interpreted. Consequently, we analyzed the association between life satisfaction and the two dimensions of friendship in a standard multilevel logistic regression setting where the dependent variable is recoded into categories, obtained using different thresholds.

Several variations on recoding have been considered to test the robustness of the model to the choice of the threshold. We considered three binary categorizations, using threshold 6 (usually conceived as “sufficiency,” since it is the mark distinguishing between pass and fail in tests at school in Italy), 7 (the mean satisfaction score in the sample), and 8, which is the threshold value used by Istat (2015, 2016). After that, the corresponding multilevel binary logistic regression was estimated. A categorization into three levels (< 6, 6, and 7, ≥ 8) was also considered and a multilevel multinomial logistic regression model was used for the estimation. This model did not reach convergence because of the high percentage (40%) of second-level units (family), including only one first-level unit (individual). In the following, only the results deriving from the multilevel binary logistic regression, which is briefly described in the following lines, were reported.

Let *N* be the number of second level units and *n*_j_ (*j = 1*,…, *N*) be the number of first level units in group *j*. Let *Y*_ij_ denote the dichotomous variable taking value 1 if the life satisfaction of an individual is at least 7 and 0 otherwise. The two outcomes are coded as “satisfied” and “not satisfied”, respectively. Variables that are potential explanations for *Y*_ij_ are denoted by *X*_1_, …, *X*_k_. Let *π*_ij_ be the probability that an individual *i* in the group *j* is satisfied. A logistic random intercept model expresses the logit of *π*_ij_ as a sum of a linear function of the explanatory variables and a random second-level (family)-dependent error *ε*_0j_:

$$ \mathrm{logit}\left({\pi}_{\mathrm{ij}}\right)={\beta}_0+{\sum}_{k=1}^p{\beta}_k{x}_{\mathrm{kij}}+{\varepsilon}_{0\mathrm{j}}, $$

where *β*_k_ are statistical parameters that need to be estimated from the data.

### Control variables

Following previous studies (see for instance Huxthold et al. 2013), other explanatory variables were included in the model to allow for consideration of the net association between life satisfaction and the two aspects of friendship. First, variables measuring potential social relations were included in the model. Results were controlled for the social integration and active lifestyle. Social integration was inserted into the models because of its importance for subjective well-being (as discussed in the “Social relations, friendship, and life satisfaction” section) and was measured considering the participation in meetings organized by political parties, trade union organizations, or by other (e.g., voluntary or cultural) associations in the year prior to the interview. Individuals who participated in at least one of these activities were distinguished from those with no participation. An active lifestyle was considered for its benefits on physical and psychological health (see, for example, Hassmén et al. 2000). It was measured using a covariate that described physical activities and distinguished individuals as follows: playing sports regularly, those engaged in physical activity at least once a week, and those who were physically active less often or who were sedentary. Attendance at religious services was also included in the model, both for the social networks that people find in religious organization and for the private and subjective aspects of religion (Lim and Putnam 2010). This control variable is defined by three categories of attendance: at least once a week, sometimes in a month or in a year, and never.

Next, the multivariate analyses are controlled for a series of covariates grouped into three main domains which the literature has shown to be important for life satisfaction (see, for example, Siedlecki et al. 2008; Meggiolaro and Ongaro 2015): socio-economic and demographic characteristics, health status, and personality traits. The socio-economic background of individuals included their age, gender, education, employment status, and their family’s economic situation and structure. Education is controlled for through a covariate with three categories: low (junior high school or lower), middle (secondary school), and high (post-secondary education). Regarding employment status, we distinguished employed^2^ individuals from those who declared themselves to be unemployed and those who were out of the labor force (housewives, students, retired people, etc.). The family economic situation is measured through a question that subjectively evaluates family economic resources. A dichotomous covariate differentiated individuals in families with poor or insufficient resources from those with very good or good resources. Family structure was investigated keeping track of both the type of family and an individual’s position in the family. Individuals were distinguished as follows: individuals who are paired with another and with children, individuals who are paired with another without children, individuals who are children in households with at least one parent, individuals who are parents in single-parent families, and all other cases.

Health status was measured considering three subjective indicators of health: limitations, self-analysis of health, and self-satisfaction with health. The first measured the presence of limitations and was based on individuals reporting any limitations on typical, day-to-day activities. These limitations were defined in three categories: severe limitations, only mild limitations, and no limitations. The second indicator was obtained by a question asking individuals how they viewed their health; the five available responses were grouped into three categories: good (excellent or good), fair, and poor (poor and very poor) health. The final subjective indicator was measured by an individual’s satisfaction with health, grouped into three categories: very satisfied, quite satisfied, and not satisfied (including individuals who declared themselves as not very satisfied or not satisfied at all).

An individual’s personality was identified through two indicators. The first was obtained from a question investigating whether individuals trust people; the results distinguished those who declared that most people can be trusted from those who thought that they must be very careful. The second indicator was obtained from a question asking individuals for their views on future and personal situations, with four response options: the situation will improve, it will remain the same, the situation will worsen, and “do not know.” In the analyses, the individuals were grouped into optimistic, pessimistic, and indifferent categories; the last merging people who did not know with those who declared the situation will remain the same. Along the same lines, an individual’s satisfaction on specific aspects of life, ranging from employment^3^ and economic resources to family relationships and leisure time, was taken into account by the model.^4^ Identical to questions on friendship relationships, the corresponding response options consisted of the following: 1 = very satisfied, 2 = quite satisfied, 3 = not very satisfied, and 4 = not satisfied at all. In the following analyses, the last two categories are grouped as one.

Finally, the results were controlled for the geographical area of residence (north-west, north-east, center, and south), and the type of municipality (distinguishing, by way of population count, metropolitan areas and suburbs from other towns) for the potential importance of economic, social, environmental, and urban development of the area in which individuals live (González et al. 2011).

Before estimating the model, associations among the explanatory variables were checked using the normalized mutual information. All the values were close to 0, thereby suggesting the absence of strong correlation among the control variables.

## Results

As described in the “Methods and strategy of analysis” section, three thresholds have been used to categorize the dependent variable and investigate the robustness of the model to the choice of the threshold. The corresponding, multilevel logistic regression models lead to the same estimated effects, thereby indicating that the model is robust to the choice of the threshold. Here, we report only the results^5^ considering the threshold value 7 (Table 3). The appropriateness of the multilevel specification to account for the data structure as revealed by the intercept variance significance should be noted.

**Table 3:** Coefficient estimates (*β*) and their standard errors (s.e.), odds ratios (OR), and their 95% confidence interval of the binary logistic multilevel model for the life satisfaction (probability of being satisfied)

	Est.	s.e.	OR	95% CI	
Intercept	6.315	0.268***			
Variance	2.765	0.179***			
Gender (ref. male)					
Female	− 0.045	0.045	0.956	0.860	1.044
Age	− 1.001	0.183***	0.367	0.257	0.526
Age (squared)	0.949	0.177***	2.584	1.829	3.650
Education (ref. high)					
Low	0.306	0.075***	1.357	1.171	1.574
Medium	0.182	0.051***	1.200	1.087	1.325
Employment status (ref. employed and very satisfied)					
Other	− 1.754	0.106***	0.173	0.141	0.213
Unemployed	− 0.912	0.098***	0.402	0.332	0.486
Not Satisfied	− 1.610	0.105***	0.200	0.163	0.246
Quite satisfied	− 0.473	0.091***	0.623	0.522	0.745
Economic resources (ref. good or very good)					
Poor or insufficient	− 0.434	0.056***	0.648	0.581	0.724
Family’s structure (ref. couples with children)					
Parents in single-parent families	− 0.670	0.102***	0.512	0.419	0.625
Couples without children	− 0.175	0.077**	0.840	0.723	0.976
Child	− 0.808	0.082***	0.446	0.380	0.523
Others	− 0.634	0.074***	0.530	0.459	0.613
Perception of health (ref. good)					
Fair	− 0.589	0.162***	0.555	0.404	0.761
Poor	− 0.505	0.063***	0.604	0.534	0.682
Presence of limitations (ref. no)					
Severe limitations	− 0.236	0.144	0.790	0.596	1.047
Only mild limitations	0.097	0.073	1.102	0.955	1.272
Health satisfaction (ref. very satisfied)					
Not satisfied	− 0.787	0.104***	0.455	0.371	0.558
Quite satisfied	− 0.330	0.065***	0.719	0.632	0.817
Attendance at religious services (ref. At least one a week)					
Never	− 0.353	0.072***	0.703	0.611	0.808
Sometimes	− 0.036	0.058	0.964	0.861	1.079
Social integration (ref. yes)					
No	− 0.289	0.055***	0.749	0.673	0.834
Sport (ref. regularly)					
Never	− 0.291	0.062***	0.747	0.662	0.844
At least one per week	− 0.001	0.065	0.999	0.880	1.134
Trust in other people (ref. yes)					
No	− 0.462	0.059***	0.630	0.562	0.707
View of personal situation in the future (ref. optimistic)					
Indifferent	− 0.549	0.056***	0.578	0.518	0.645
Pessimistic	− 1.150	0.070***	0.317	0.276	0.364
Leisure time satisfaction (ref. very satisfied)					
Not satisfied	− 0.531	0.084***	0.588	0.499	0.694
Quite satisfied	− 0.115	0.079	0.891	0.763	1.041
Area of residence (ref. north-west)					
South	− 0.204	0.072***	0.815	0.709	0.938
Center	− 0.273	0.082***	0.761	0.648	0.894
North-east	− 0.154	0.080**	0.857	0.733	1.004
Type of municipality (ref. metropolitan area)					
> 50,000	− 0.037	0.099	0.963	0.793	1.170
Town with 10,000–50,000	0.044	0.091	1.045	0.874	1.249
Town with 2000–10,000	0.267	0.093***	1.305	1.090	1.564
Town with less than 2000 inhabitants	0.238	0.1184**	1.268	1.006	1.600
Suburbs	− 0.155	0.116	0.856	0.683	1.074
Economic resources satisfaction (ref. very satisfied)					
Not satisfied	− 1.518	0.206***	0.219	0.146	0.328
Quite satisfied	− 0.357	0.205	0.700	0.468	1.046
Family relationships satisfaction					
Not satisfied	− 1.526	0.105***	0.217	0.177	0.267
Quite satisfaction	− 0.681	0.061***	0.506	0.450	0.570
Frequency of meeting friends (ref. more than once a week)					
Only some times a year or without friends	− 0.306	0.083***	0.737	0.626	0.866
Once a week or several times a month	− 0.087	0.051*	0.916	0.829	1.013
Friendship relationships satisfaction (ref. very satisfied)					
Quite satisfied	− 0.519	0.094***	0.595	0.495	0.716
Not satisfied	− 0.301	0.068***	0.740	0.648	0.846

Significant parameter at *p < .1, **p < .05, ***p < .01

The demographic characteristics are considered first. Gender is not significant, suggesting that there are no differences in life satisfaction between men and women. The parameters associated with the age are both significant, suggesting that there is a quadratic relation between life satisfaction and age. The linear combination of the estimates indicates that the oldest people tend to be more satisfied than the youngest. It is observed that individuals with a high level of education tend to be less satisfied than those possessing a lower or medium level of education. These results can be related to the different expectations the young (more eager for life) and, to some extent, more educated people (more acute in the evaluation of their living conditions) have with respect to those who are older and less educated. Regarding the age effect, the difference with the aforementioned literature might be due to a diverse context of analysis and/or to the choice of other control variables.

The coefficients of the variables related to the economic status show that employed people (particularly those who declared to be very satisfied with their work) with adequate economic resources tend to be more satisfied than the others. The coefficients related to the family’s structure suggest that individuals living in couples (with or without children) tend to be more satisfied with their life when compared to people living in other family structures.

Social integration and active lifestyle, with all its aspects, also play an important role. The more integrated an individual is, the more satisfied he/she is, as suggested by the positive coefficient related to social integration. The model estimates also suggest that people attending religious services (regularly or sometimes in a year) tend to be more satisfied with their life than people not attending religious services. A similar result is observed for physical activities, where a moderate physical activity leads to higher life satisfaction. The negative coefficients of the health status, measured by the individual subjective perception indicate that a worse health status correlates to a lower life satisfaction. Similarly, the coefficients related to the presence of limitations indicate that individuals with severe limitations tend to be less satisfied than those who do not have limitations.

An individual’s personality traits also affect life satisfaction. Trusting other people and having a positive attitude increase the probability of having high life satisfaction. Similarly, the data suggests that an individual’s high satisfaction with facets of their life (economic, health and family relationships, and free time) correlates to a higher life satisfaction.

Finally, the coefficients related to variables concerning the geographical area individuals live in suggest that living in the north-west area increases the probability of being satisfied. For the type of municipality, the model suggests that individuals living in a town with more than 2000 inhabitants, but less than 10,000, have a higher probability of being satisfied.

The coefficients related to the key variables showed that friendship relationships were associated with life satisfaction. In particular, the probability of an individual who meets friends once a week or several times a month being satisfied with life is 9% lower than the same probability for an individual who meets his friends regularly. If the individual meets friends only a few times a year or does not have friends, then the probability of being satisfied decreases nearly 27%. Moreover, if individuals are either quite satisfied or not satisfied with their friendship relationships, then the probability of being satisfied decreases 49 and 69%, respectively, compared to the same probability for individuals satisfied with their friendship relations. We also tested the presence of several interaction effects. First, a synergy effect between the frequency of meeting friends and the friendship satisfaction was checked for. This enabled testing if frequent and satisfactory friendship relations might increase the probability of being satisfied with life. The corresponding parameters turned out to be not significant.

In addition, the interaction between type of municipality and friendship satisfaction and intensity of friendship, respectively, was considered. The motivation relies on the fact that many network studies (e.g., Adams et al. 2012), aiming at defining the effect of the geographical space on the configuration of the network, have suggested that smaller areas and proximity facilitate contacts and are contexts where people get to know each other more easily. The analysis indicated that only the interaction between being not satisfied and living in a small area was negative and significant. Since all the other interactions were not significant, and the conclusions for all the other variables did not change when including or excluding interactions, only the more parsimonious models without interactions are reported in the paper.

## Concluding remarks

The analysis of social capital focuses on the set of relationships in which individuals are embedded. These relations are resources for the individuals themselves and might have an impact on some aspects of their life, e.g., performance, well-being, and support.

An analysis of a particular facet of social capital, namely the role of friendship relations on the life satisfaction of people aged 18–65, was conducted. Using data from the multipurpose survey “Aspects of daily life,” collected by the Italian National Statistical Institute in 2012, a multilevel logistic model was estimated to study the link between life satisfaction and the frequency of meeting friends, as well as the satisfaction with friendship relationships. This link is considered, by psychological literature, as a bidirectional dynamic process (Demir et al. 2015). Having friends and close peer experiences are both important predictors of life satisfaction, and satisfied individuals tend to have stronger and more intimate social relationships.

Although in the current study the target variables follow a partially logical chronological order, the data derives from an observational study, and therefore, no causal relations can be inferred. Consequently, we only focused on the association between life satisfaction and friendship controlling for all other potential confounding variables that we have at disposal. This is a limitation of the study that may only be addressed using longitudinal data.

The results of the analysis showed that less frequent meetings contributed to lower friendship relationship satisfaction, thus leading to lower life satisfaction. These findings were robust to the choice of different thresholds and to a wide set of control variables—with significant associations—pertaining to three main domains that literature has shown to affect life satisfaction.

The current study supports the finding that friends are relevant nodes in a personal network. A high life satisfaction is indeed associated with the presence of friendship. This might be explained by the positive functions attributed to friends. As suggested by previous research, friends provide companionship (in addition to more social trust and less stress), intimacy, and help, which increase an individual’s life satisfaction (see, for example, Demir and Weitekamp 2007).

Furthermore, the results indicate that both having/meeting friends and good-quality friendship relations are important to an overall life satisfaction. Individuals may benefit from positive interactions with friends, which are a part of an individual’s social capital. High-quality friendships are more likely to be characterized by support, reciprocity, and intimacy. Conversely, low-quality relations and/or the lack of positive interaction may elicit anxiety.

The importance and the impact of friendship on the life of individuals indicate that it is worthwhile to deepen the topic of friendship relationships and the “contexts in which such relationships are embedded” (Adams and Allan 1998). A study of the impact could also be beneficial in population studies. Like all other types of personal relationships, friendships are indeed “constructed-developed, modified, sustained, and ended—by individual acting in contextual setting” (Adams and Allan 1998, p.3), which is defined by age, gender, stage of life, living arrangement, and experiences lived. These settings might affect the mechanisms of friendship formation and characterization in different ways and, consequently, the measurement of quantitative and qualitative dimensions of friendships.

## Footnotes

1 This categorization has been suggested by preliminary analyses which considered all the seven categories and showed not significant differences between categories 1 and 2, between 3 and 4, and across categories 5, 6, and 7.

2 For employed individuals, also their satisfaction with work is considered, distinguishing those very satisfied, those quite satisfied, and those not satisfied; this follows the perspective suggested below to consider also the individuals’ satisfaction with different aspects of their life.

3 As mentioned above, satisfaction with work is embedded in the variable describing employment status (see footnote 2).

4 There might be a reversed relationship between life satisfaction and satisfaction in the different domains of individual’s life. While on the one hand, satisfaction in domains of life might affect life satisfaction; on the other hand, overall life satisfaction might affect individual’s satisfaction. The issue of reverse causality has been discussed in the literature starting from the distinction between top-down and bottom-up theories of well-being by Diener (1984) and has not yet been settled by the empirical research (see, for example, the discussion in Møller and Saris 2001; Rojas 2006; González et al. 2010). In the current analysis, we aim only at investigating the association between satisfaction in the different domains of individual’s life and life satisfaction. The study of bidirectional causal relation between life satisfaction and friendship relations is beyond the aims of the current paper. In fact, probably with the current data, there is not the problem of reversed relationship between satisfaction in the different domain of individual’s life and life satisfaction; in the questionnaire, indeed, the time reference of the different domains of individual life was the last 12 months, whereas the evaluation of life satisfaction was referred to the “usual” or “normal” behavior of the respondents without a precise time reference, and thus the former aspects are referred to a time that preceded the time reference of life satisfaction.

5 The models were estimated using the procedure GLIMMIX in the program SAS.
